# Efficacy of Seed-Biopriming with *Trichoderma* spp. and Foliar Spraying of ZnO-Nanoparticles Induce Cherry Tomato Growth and Resistance to *Fusarium* Wilt Disease

**DOI:** 10.3390/plants12173117

**Published:** 2023-08-30

**Authors:** Amany H. M. Shams, Amira A. Helaly, Abeer M. Algeblawi, Eman F. A. Awad-Allah

**Affiliations:** 1 Plant Pathology Department, Faculty of Agriculture, Alexandria University, Alexandria 21545, Egypt; amany.shams@alexu.edu.eg; 2 Vegetable Crops Department, Faculty of Agriculture, Alexandria University, Alexandria 21545, Egypt; amira.helaly@alexu.edu.eg; 3 Plant Protection Department, Faculty of Agriculture, University of Tripoli, Tripoli 13479, Libya; a.algeblawi@uot.edu.ly; 4 Soil and Water Sciences Department, Faculty of Agriculture, Alexandria University, Alexandria 21545, Egypt

**Keywords:** *Fusarium solani*, plant immunity, plant nano-nutrition, seed-biopriming, sustainability, volatilomics

## Abstract

Several microbes that cause plant diseases drastically lower the production of agriculture and jeopardize the safety of the world’s food supply. As a result, sustainable agriculture requires disease management tactics based on modern, eco-friendly techniques as alternatives to various agrochemicals. The current study aimed to assess the antifungal activity of ZnO-nanoparticles against *Fusarium solani* in-vitro, and the ability of two antagonistic *Trichoderma* isolates, *Trichoderma viride* and *Trichoderma harzianum*, to produce antifungal secondary metabolites and identify them using gas chromatography–mass spectrometry, and to evaluate the combined effects of foliar spray of ZnO-nanoparticles and bioprimed seeds of cherry tomato (*Solanum lycopersicum* L.) with two antagonistic *Trichoderma* isolates against *Fusarium* wilt disease caused by *Fusarium solani* in greenhouse conditions. The results revealed that, in-vitro, the highest concentration of ZnO nanoparticles (3000 ppm) resulted in the greatest decrease in *Fusarium solani* mycelial growth (90.91% inhibition). The scanning electron microscopy demonstrated the evident distortion in *Fusarium solani* growing mycelia treated with ZnO-nanoparticles, which might be the source of growth suppression. Additionally, twenty-eight bioactive chemical compounds were isolated and identified from *Trichoderma* spp. ethyl acetate crude extracts using gas chromatography–mass spectrometry. In a greenhouse experiment, the combination of bioprimed cherry tomato plants with *Trichoderma harzianum* and foliar spraying of ZnO-nanoparticles at 3000 ppm was the most effective interaction treatment for reducing disease severity index (23.4%) and improving the vegetative growth parameters, micronutrient contents (Mn, Zn, and Fe in leaves), and chlorophyll content (SPAD unit), as well as stimulating phenylalanine ammonia-lyase activity of cherry tomato leaves at 75 days after sowing. In conclusion, the antifungal potential of seed-biopriming with antagonistic *Trichoderma* isolates and the foliar spraying of ZnO-nanoparticles can boost cherry tomato growth and confer resistance to *Fusarium* wilt caused by *Fusarium solani*.

## 1. Introduction

Plants are susceptible to a range of biotic stressors caused by many organisms, which result in a variety of diseases, infections, and crop plant damage, eventually impacting agricultural productivity [1]. *Fusarium* wilt, for example, is a devastating disease produced by fungal soil-borne pathogens such as *Fusarium oxysporum* and *Fusarium solani* that causes significant losses for many essential vegetable and agricultural plants, especially tomatoes, in Egypt and throughout the world [2]. In several Egyptian governorates, *F. solani* isolates were the most frequently found soil-borne pathogenic fungus in tomato plants, causing damping-off and root rot illnesses [3]. Infected tomato plants first exhibit stunted seedlings and yellowing of older leaves, followed by wilting that progresses up the stem until the foliage falls off and brown vascular coloring in cross sections of stem tissue near the soil line [2]. Because there is no spore development above ground in the field, *Fusarium* wilt does not transfer from plant to plant during a season, but it can be disseminated through contaminated, infected seeds [4,5]. Biological seed treatments with effective fungal or bacterial biocontrol agents may provide an alternative to chemical control of many soil- and seed-borne pathogens [6,7]. Biopriming is an innovative and sustainable approach to seed treatment that combines biological (inoculation of the seed with beneficial organisms to protect it) and physiological (seed hydration) aspects of disease prevention [8]. Consequently, biopriming improves nutrition and water intake, increases seedling vigor, and establishes systemic resistance to biotic and abiotic stressors [9]. In recent years, several *Trichoderma* spp., such as *T. viride* and *T. harzianum*, have been commonly utilized as effective biocontrol agents, biofertilizers, and plant growth boosters in biotic and abiotic stress conditions [6,10,11]. However, it is necessary to investigate their ability to reduce the severity of *Fusarium* wilt disease if they are applied as a biopriming treatment. Additionally, *Trichoderma* spp. have been shown to generate different arrays of soluble and volatile organic compounds (VOCs) against rhizosphere pathogens, implying that they are an important source of biologically active natural metabolites [6,9,12]. Therefore, mining for the volatilomes of plant-associated microbiota for new biocontrol solutions is necessary [13]. The use of VOCs may possibly be an ecological and safe strategy for managing plant diseases and accelerating the transition to a more sustainable food production system [13].

Complete and balanced plant nutrition has always been the first line of defense for plants, because mineral elements are directly engaged in plant immunity and protection as structural components and metabolic regulators [14,15,16]. As a result, the nutritional status of plants can affect their vulnerability or resistance toward fungal pathogen-caused diseases [15,16]. Zinc (Zn), for example, is a part of metalloenzymes that are involved in auxin synthesis, infectivity, phytotoxin, and mycotoxin production in pathogenic microorganisms [16]. The efficient use of zinc oxide nanoparticles (ZnO-NPs) as plant nano-nutrition is rapidly increasing, which can help alleviate both biotic and abiotic stress, providing novel solutions to plant and agricultural science challenges [14,17]. However, no research has been conducted on the effect and mode of action of ZnO-NPs on the growth of the soil-borne fungus *F. solani*. Also, the use of ZnO-NPs alone or in combination with antagonistic *Trichoderma* isolates against *F. solani* has not been studied. Therefore, the objectives of the current research were: (1) to evaluate the antifungal activity of ZnO-NPs against *F. solani* in-vitro; (2) to examine the ability of two antagonistic *Trichoderma* isolates, such as *T. viride* (*T*_v_) and *T. harzianum* (*T*_h_), to create antifungal secondary active metabolites and identify them using gas chromatography–mass spectrometry (GC–MS) analysis; and (3) to assess the combined effects of foliar spray of ZnO-NPs and bioprimed cherry tomato seeds with two antagonistic *Trichoderma* isolates (*T*_h_ and *T*_v_) against *Fusarium* wilt disease caused by *F. solani* in greenhouse conditions, as well as to assess their antifungal activity efficacy in reducing the severity of *Fusarium* wilt disease and increasing vegetative growth and development of cherry tomatoes.

## 2. Results

### 2.1. Evaluation of the Antifungal Activity of ZnO-NPs against F. solani In-Vitro

#### 2.1.1. Culture Medium Amended with ZnO-NPs

**Figure 1:**
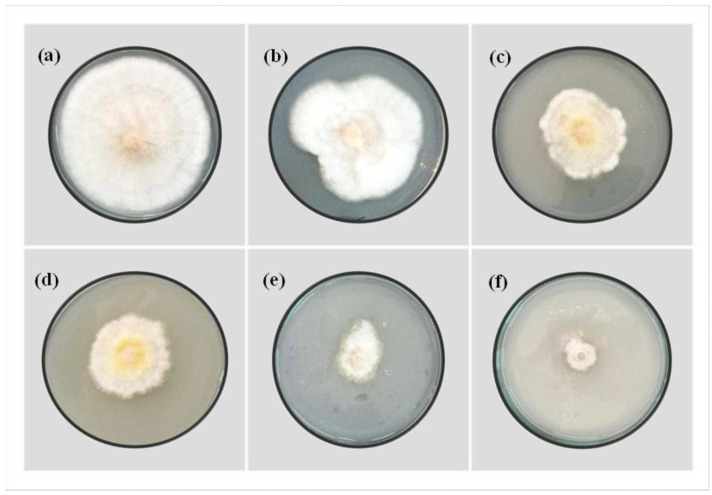
Antifungal activity of ZnO-NPs on *F. solani* incubated for 12 days at 25 ± 2 °C in-vitro test: (**a**) 0 ppm, (**b**) 250 ppm, (**c**) 500 ppm, (**d**) 1000 ppm, (**e**) 1500 ppm, (**f**) 3000 ppm.

The colony diameter of *F. solani* significantly (*p* ≤ 0.05) decreased with increasing ZnO-NP concentrations, as shown in Figure 1 and Table 1. The use of a ZnO-NP suspension efficiently inhibited the fungal growth of *F. solani* (Figure 1). After 12 days, the average mycelial growth inhibition of *F. solani* has ranged from 17.05 ± 3 to 90.91 ± 2%, as ZnO-NP concentration increased from 250 to 3000 mg L^−1^. These findings imply that ZnO-NPs can disrupt and damage the fungal conidia. These results highlight ZnO-NPs’ significant potential as antifungal agents that can be used to control the spread of *F. solani*, as well as their ability to reduce the environmental impact of synthetic fungicides.

#### 2.1.2. Scanning Electron Microscopy (SEM) Analysis

**Figure 2:**
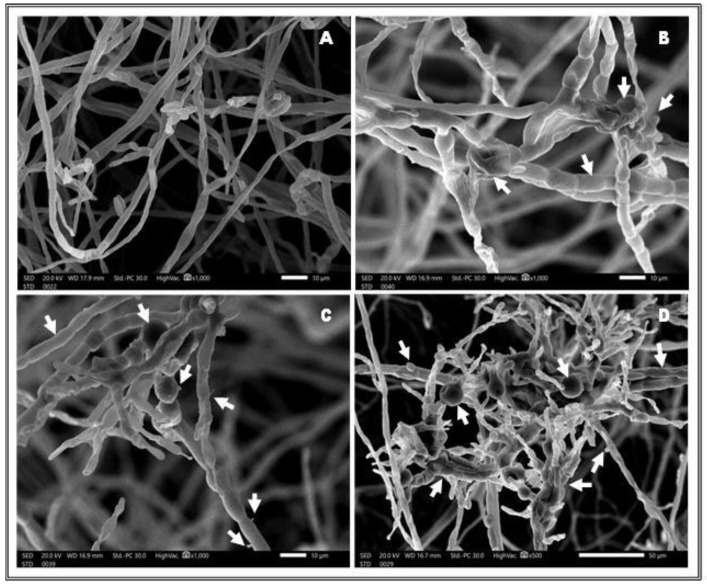
SEM micrograph of growing *F. solani* (**A**) ZnO-NP-free (control), and (**B**–**D**) ZnO-NPs (3000 ppm). *F. solani* cultures were incubated for 12 days at 25 ± 2 °C. White arrows point to the structural alterations caused by nanoparticle treatment.

In order to evaluate the effects of ZnO-NPs on the *F. solani* pathogen, SEM analysis was used to determine the structural alterations caused by nanoparticle treatment. Figure 2 compares images of mycelia obtained from the edge of *F. solani* cultures inoculated over PDA containing 3000 mg L^−1^ ZnO-NPs to a control containing no ZnO-NPs during a 12-day incubation period at 25 ± 2 °C. The control sample (ZnO-NP-free) included hypha with a typical net structure and smooth surface before being treated with ZnO-NPs (Figure 2A). After 3000 mg L^−1^ ZnO-NPs treatment, the surface of *F. solani* had a rough texture with the growth of mycelia clusters, the formation of protrusions, and the appearance of swollen and coiled hyphae (Figure 2B–D). This suggested that *F. solani* growth inhibition mediated by ZnO-NPs might be related to deformation and morphological aberrations in the structure of the fungal hypha.

### 2.2. Identification of Secondary Metabolites of Trichoderma Isolates by Gas Chromatography–Mass Spectrometry Analysis:

**Figure 3:**
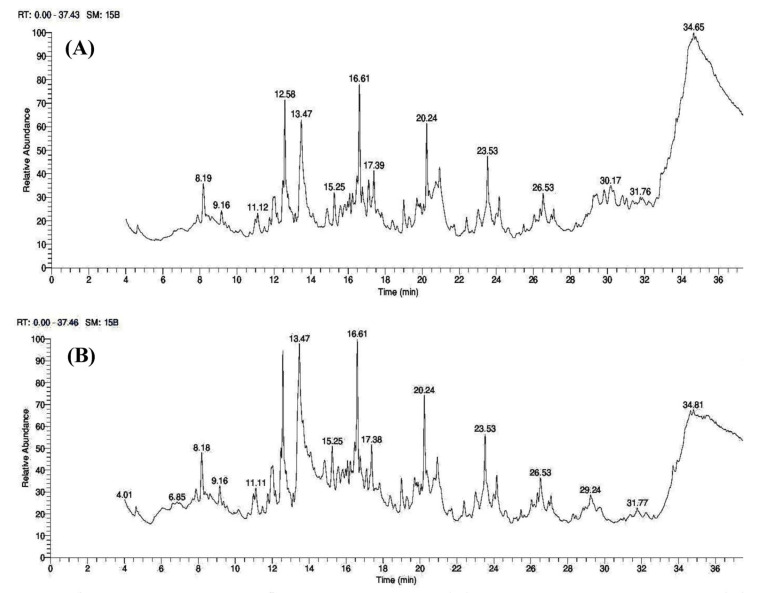
The GC–MS mass spectra of identified bioactive compounds from crude extracts of (**A**) *T. viride*, (**B**) *T. harzianum*.

**Figure 4:**
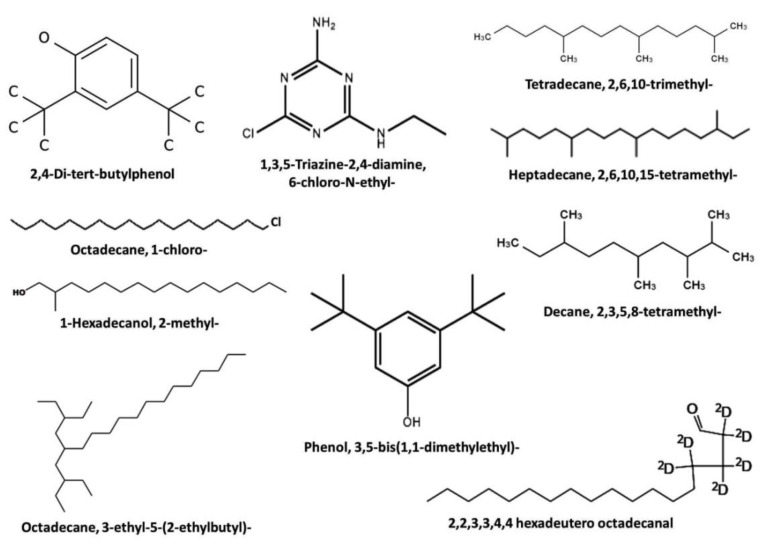
Chemical structures of the ten most abundant (%) bioactive chemical compounds detected by GC–MS analysis of ethyl acetate extracts of culture filtrate of *Trichoderma* sp.

GC–MS analysis was used to identify the bioactive compounds in the crude extracts of *Trichoderma* isolates, as shown in Figure 3 and Figure 4. A total of twenty-eight bioactive chemical compounds were extracted and identified from *Trichoderma* spp. ethyl acetate crude extracts, all of which had substantial antimicrobial and growth-stimulating activities against rhizosphere pathogens, according to several studies listed in Table 2**.** As a result, they can protect cherry tomatoes from infections, confer resistance, and boost development in cherry tomatoes.

The analyzed data revealed the presence of a high relative abundance of Tetradecane, 2,6,10-trimethyl- (21.70% *T*_v_, and 22.88% *T*_h_); Heptadecane,2,6,10,15-tetramethyl- (14.11% *T*_v_, and 20.99% *T*_h_); 1,3,5-Triazine-2,4-diamine,6-chloro-N-ethyl- (16.02% *T*_v_, and 18.87% *T*_h_); Octadecane,1-chloro- (11.57% *T*_v_, and 16.28% *T*_h_); Decane,2,3,5,8-tetramethyl- (9.48% *T*_v_, and 13.07% *T*_h_); Octadecane,3-ethyl-5-(2-ethylbutyl)- (5.91% *T*_v_, and 8.02% *T*_h_); 2,4-Di-tert-butylphenol (5.80% *T*_v_, and 7.09% *T*_h_); 2,2,3,3,4,4 hexadeutero octadecanal (5.50% *T*_v_, and 7.03% *T*_h_); Phenol, 3,5-bis(1,1-dimethylethyl)- (4.73% *T*_v_, and 5.85% *T*_h_); 1-Hexadecanol, 2-methyl- (4.26% *T*_v_, and 5.18% *T*_h_), as shown in Table 2 and Figure 4.

### 2.3. Effect of Seed-Biopriming with Trichoderma and ZnO-NPs against Tomato Fusarium Wilt Disease under Greenhouse Conditions

#### 2.3.1. Disease Severity Index (%)

**Figure 5:**
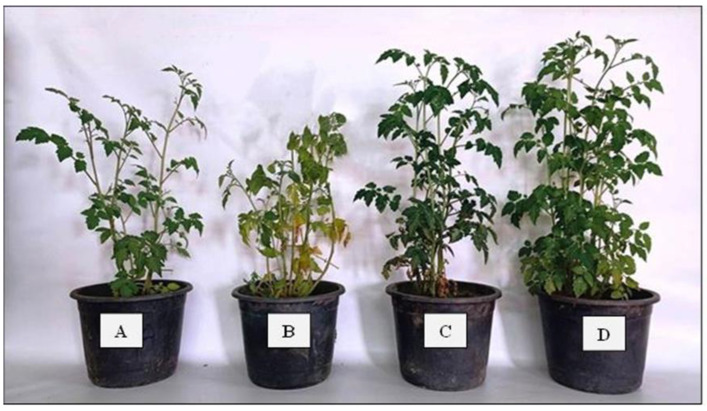
Infected cherry tomato at 75 DAS, as affected by different treatments of seed-biopriming with *Trichoderma* isolates and foliar spray of ZnO-NPs. (**A**) Non-infected control, (**B**) infected control “*F. solani*”, (**C**) infected cherry tomato with *T. viride* + (ZnO-NPs)_3000 ppm_, and (**D**) infected cherry tomato with *T. harzianum* + (ZnO-NPs)_3000 ppm_.

Cherry tomato plants infected with *F. solani* had the highest mean disease severity index (92.6%), according to Table 3. As illustrated in Figure 5B, untreated, infected control plants displayed stunted seedlings, yellowing of older leaves, wilting, root rot, and a shorter main root as compared with absolute healthy control plants (Figure 5A). In comparison to untreated, infected control plants, bioprimed cherry tomato plants with various *Trichoderma* isolates combined with an exogenous foliar spray of ZnO-NPs (ppm) significantly (*p* ≤ 0.01) reduced the *Fusarium* wilt disease severity index (%). Furthermore, our findings demonstrated that the interaction between bioprimed cherry tomato plants with *T. harzianum* (*T*_h_) and foliar sprays of ZnO-NPs at 3000 ppm was the most effective interaction treatment for reducing cherry tomato disease severity index (23.4%). The suppressive effects of foliar spraying of ZnO-NPs (3000 ppm) and seed-biopriming treatment with *T. harzianum* (*T*_h_) against *Fusarium* wilt disease are shown in Figure 5D. In addition, these treated plants looked healthy, with no symptoms of *Fusarium* wilt disease (Figure 5D). Figure 5C depicts the effects of ZnO-NPs (3000 ppm) and bioprimed cherry tomato with *T. viride* treatments on *Fusarium* wilt disease. However, the bioprimed cherry tomato with *T. harzianum* isolate was more resistant to the *F. solani* pathogen than the bioprimed cherry tomatoes with *T. viride* isolate when combined with a foliar spray of ZnO-NPs at 3000 ppm, as shown in Table 3 and Figure 5. These findings suggest that using ZnO-NPs alone or in combination with antagonistic *Trichoderma* isolates can give better protection against *F. solani* infection to cherry tomato plants.

#### 2.3.2. Vegetative Growth Parameters

**Figure 6:**
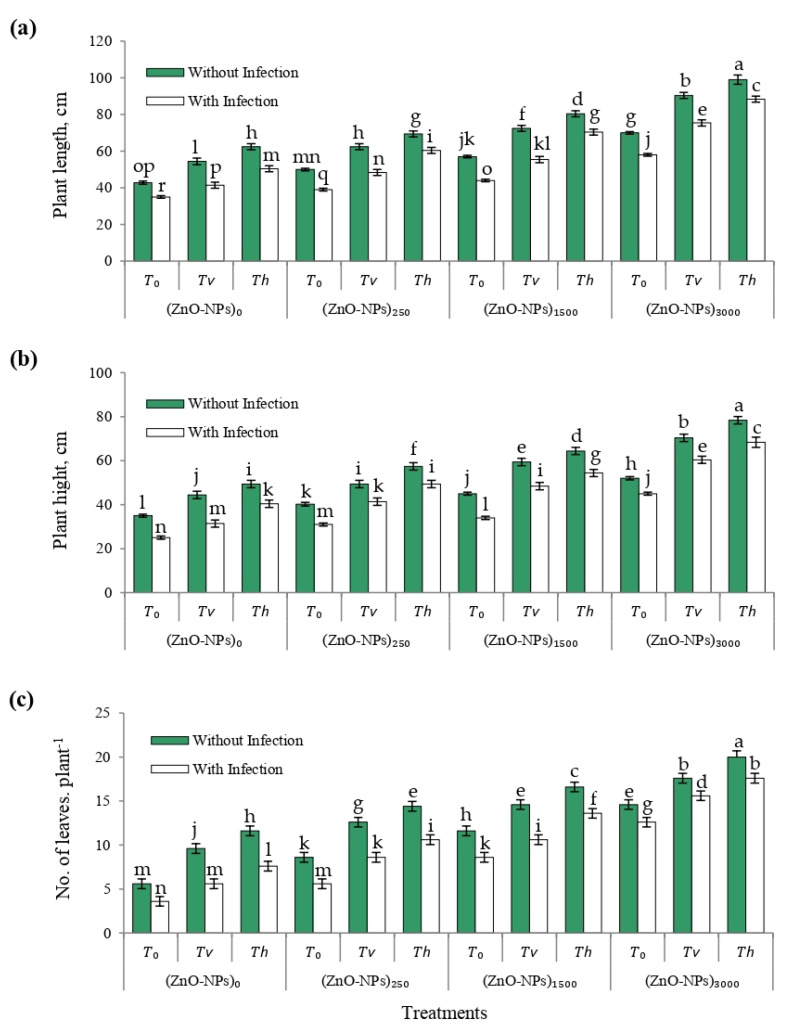
Vegetative growth parameters of cherry tomato plants at 75 DAS, as affected by different treatments of seed biopriming with *Trichoderma* isolates, and foliar spray of ZnO-NPs with and/or without *F. solani*-infested soil. (**a**) plant length, (**b**) plant height, and (**c**) number of leaves per plant. Error bars represent the mean ± standard deviation (±SD) of the data of 5 replications. Different letter(s) above the error bars indicate statistically significant differences at (*p* ≤ 0.05).

On the studied vegetative growth parameters of cherry tomato plants, such as plant length, plant height, and number of leaves per plant, Figure 6 depicts the effects of various treatments involving seed-biopriming with *Trichoderma* isolates and foliar spray of ZnO-NPs alone, as well as their interactions with and/or without *F. solani*-infested soil. The obtained results revealed that vegetative growth parameters of bioprimed cherry tomatoes with both *Trichoderma* species were significantly (*p* ≤ 0.05) increased alone and in combination with increasing ZnO-NP levels with and/or without *F. solani*-infested soil. Also, after foliar spraying with various ZnO-NP levels, bioprimed cherry tomato seeds cultivated in *F. solani*-infested soil were reported to elicit significant recovery from the reduction in vegetative growth parameters induced by wilt disease. The interaction effect of foliar sprays of ZnO-NPs (ppm) and seed-biopriming treatments on different vegetative growth parameters was significantly greater than the control treatment with and/or without *F. solani*-infested soil. In this regard, cherry tomato plants that were sprayed with ZnO-NPs (3000 ppm) and bioprimed with *T. harzianum* (*T*_h_) resulted in the greatest values of plant length, plant height, and number of leaves per plant at 75 DAS under greenhouse conditions.

#### 2.3.3. Mineral Content in Cherry Tomato Leaves

**Figure 7:**
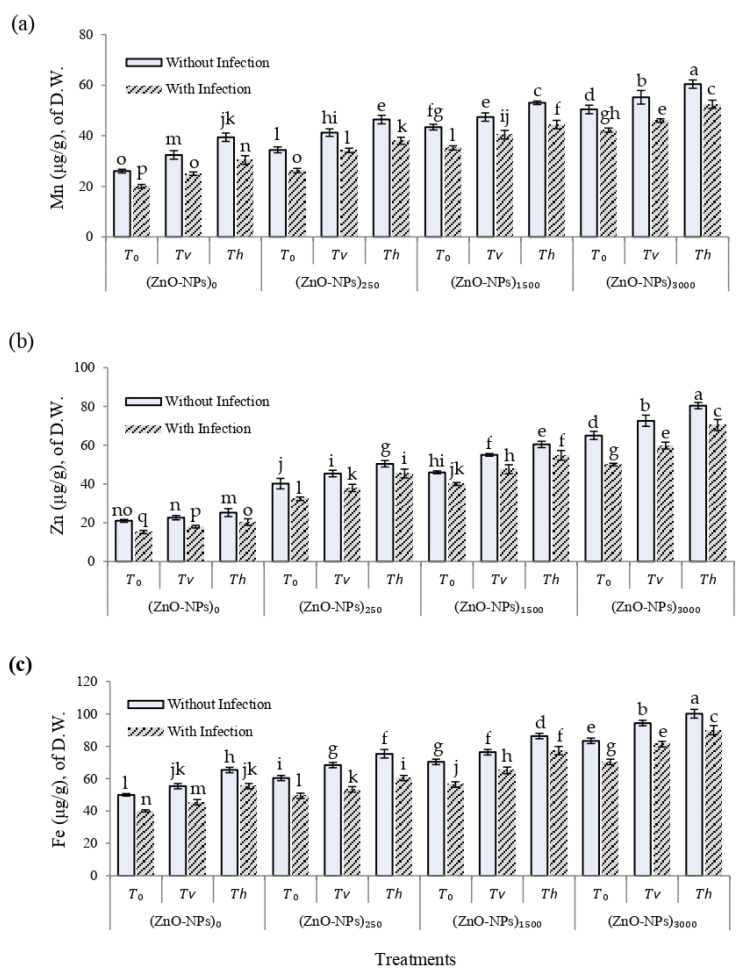
Leaf chemical composition (μg/g) of D.W. in cherry tomato plants at 75 DAS, as affected by different treatments of seed-biopriming with *Trichoderma* isolates, and foliar spray of ZnO-NPs with and/or without *F. solani*-infested soil. (**a**) Mn, (**b**) Zn, and (**c**) Fe. Error bars represent the mean ± standard deviation (±SD) of the data of 5 replications. Different letter(s) above the error bars indicate statistically significant differences at (*p* ≤ 0.01).

The effects of different treatments of seed-biopriming with *Trichoderma* isolates and foliar spray of ZnO-NPs with and/or without *F. solani*-infested soil was also investigated on the leaf chemical composition (μg/g) of the D.W. of cherry tomato plants at 75 DAS (Figure 7). Some micronutrient element contents of bioprimed cherry tomato leaves, such as Mn, Zn, and Fe, were significantly (*p* ≤ 0.01) impacted in sprayed plants with different treatments of ZnO-NPs (ppm) with and/or without *F. solani*-infested soil than the control treatment. In this regard, bioprimed cherry tomato plants sprayed with ZnO-NPs (3000 ppm) with and/or without *F. solani*-infested soil exhibited higher Mn, Zn, and Fe concentrations in their leaves than the control treatment after 75 DAS. Our results show that foliar application of ZnO-NPs to bioprimed cherry tomato plants lowers disease development and improves plant growth, probably through improved mineral nutrition and host defense.

#### 2.3.4. Leaf Chlorophyll Content (SPAD Value)

**Figure 8:**
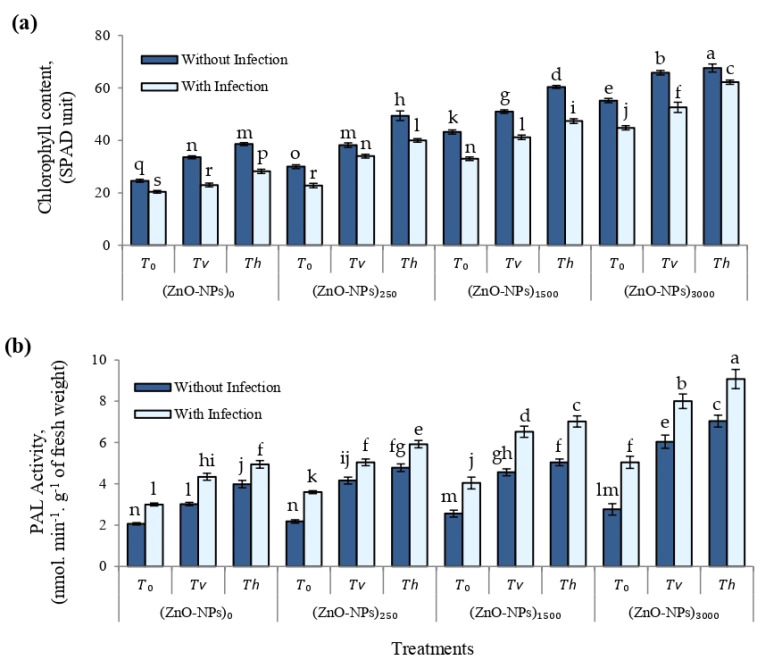
(**a**) Chlorophyll content (SPAD unit), and (**b**) phenylalanine ammonia-lyase (PAL) activity (nmol. min^−1^·g^−1^ of fresh weight) of cherry tomato leaves at 75 DAS, as affected by different treatments of seed-biopriming with *Trichoderma* isolates, and foliar spray of ZnO-NPs with and/or without *F. solani*-infested soil. Error bars represent the mean ± standard deviation (±SD) of the data of 5 replications. Different letter(s) above the error bars indicate statistically significant differences at (*p* ≤ 0.01).

Figure 8a shows the chlorophyll content (SPAD unit) of cherry tomato plants grown in greenhouse conditions as affected by different treatments of seed-biopriming with *Trichoderma* isolates and foliar spraying of ZnO-NPs with and/or without *F. solani*-infested soil. Different treatments significantly (*p* ≤ 0.01) enhanced chlorophyll content (SPAD unit) compared to infected or non-infected controls, regardless of the presence or absence of *F. solani* infection.

#### 2.3.5. Phenylalanine Ammonia-Lyase (PAL) Activity

The impact of various ZnO-NP (ppm) treatments and seed-biopriming with *Trichoderma* isolates with and/or without *F. solani*-infested soil on PAL activity (nmol. min^−1^·g^−1^ of fresh weight) of cherry tomato leaves at 75 DAS was also examined (Figure 8b). The results showed that different treatments can significantly (*p* ≤ 0.01) increase plant defense enzyme activity (such as PAL activity) in cherry tomato leaves while simultaneously enhancing cherry tomato plant growth. The combination of foliar spraying of ZnO-NPs (3000 ppm) and a seed-biopriming treatment with *T. harzianum* (*T*_h_) was the most effective treatment for stimulating PAL activity and, therefore, reducing the disease severity index (%) of cherry tomato leaves at 75 DAS. Plant defense enzyme activity, such as PAL activity, appears to improve plant resistance to rhizosphere pathogens, and may play a key role in cherry tomato defense mechanisms against *Fusarium* wilt disease caused by the soil-borne fungus *F. solani*. Our findings indicated that foliar spraying of ZnO-NPs, in combination with seed-biopriming of antagonistic *Trichoderma* isolates, might be employed as an efficient resistance strategy in cherry tomatoes for control of *Fusarium* wilt disease caused by the soil-borne fungus *F. solani*.

## 3. Discussion

Nanotechnology has the potential to significantly boost agricultural output and food security by providing environmentally acceptable alternatives to traditional agrochemicals [17]. The current study found that the in-vitro antifungal activity of ZnO-NPs can disrupt and damage *F. solani* fungal conidia. As a result, fungal growth in-vitro was significantly inhibited. The mode of action of the 3000 ppm ZnO-NP treatment was disclosed by SEM investigation, which revealed that the surface of *F. solani* had a rough texture with the growth of mycelia clusters, the generation of protrusions, and the emergence of inflated and coiled hyphae. This indicated that *F. solani* growth inhibition caused by ZnO-NPs might be linked to fungal hyphal deformation and morphological abnormalities. These findings indicate that ZnO-NPs could be employed as fungicides in agricultural and food safety applications. According to Sardella et al. [64], ZnO-NPs (size < 50 nm) have high antifungal activity, making them a suitable option for usage as a routine antifungal compound for fruit rotting prevention. Furthermore, SEM analysis demonstrated that ZnO-NPs cause substantial physical damage and evident morphological abnormalities to fungal structures, which are presumed to be permanent.

Seed-biopriming is a new productive technique for the judicious use of antagonistic bioagents to increase crop productivity under stress conditions [9]. Biopriming promotes the colonization, proliferation, and establishment of antagonistic bioagents on the seed surface [65]. Consequently, it improves the first stage of plant growth by increasing seed germination and providing protection prior to seedling emergence [8]. Therefore, it will increase seedling vigor and produce systemic resistance to biotic and abiotic stressors [9]. *Trichoderma* spp. is a common biocontrol agent that promotes plant development while inhibiting phytopathogens [9]. According to Awad-Allah et al. [10], *T. viride* and *T. harzianum* are antagonistic to the *F. solani* pathogen, which causes *Fusarium* wilt disease in cherry tomatoes. Both *Trichoderma* isolates show in-vitro mycoparasitic activity against *F. solani*, according to a dual culture test [10]. However, *T. harzianum* reduced the mycelial growth of *F. solani* by 78.0%, whereas *T. viride* inhibited the growth by 61.2% at 10 days post-inoculation through a dual culture assay [10]. Moreover, the *T. harzianum* isolate showed greater inhibition against the *F. solani* pathogen than the *T. viride* isolate through a dual culture assay [10]. According to our investigation, and the numerous research studies listed in Table 2, antagonistic *Trichoderma* isolates can produce a wide range of bioactive chemical compounds that have antifungal and plant growth-promoting activities against *Fusarium* wilt, making them a potentially suitable candidate for improving cherry tomato growth and production. The most abundant bioactive chemical compounds detected by GC–MS analysis of *Trichoderma* spp. ethyl acetate extracts were Tetradecane, 2,6,10-trimethyl-; 2,2,3,3,4,4 hexadeutero octadecanal; Octadecane,1-chloro-; Heptadecane,2,6,10,15-tetramethyl-; Decane,2,3,5,8-tetramethyl-; 2,4-Di-tert-butylphenol; Phenol, 3,5-bis(1,1-dimethylethyl)-; 1,3,5-Triazine-2,4-diamine,6-chloro-N-ethyl-; Octadecane,3-ethyl-5-(2-ethylbutyl)-; and 1-Hexadecanol, 2-methyl-. Furthermore, the majority of these bioactive compounds exhibited growth-stimulating, nutritive, and antioxidant activities, as well as acting as an elicitor for systemic acquired resistance, indicating that they can confer resistance and promote growth in cherry tomatoes, as shown by our recent study and the studies included in Table 2. Furthermore, according to Zhang et al. [31], *Trichoderma* biofertilizer application increased soil antifungal compounds that may suppress pathogenic fungus, thereby contributing to better grassland biomass. Therefore, our recent study suggested that employing volatile compounds to manage *Fusarium* wilt disease is a promising strategy, since it is a cost-effective, non-toxic, and environmentally friendly method that produces excellent long-term crop yields.

Plant nano-nutrition is a branch of plant nutrition that intends to employ nano-nutrients to provide grown plants with essential nutrients for their growth and productivity while minimizing negative environmental impacts [1,17]. The tiny size, larger surface area, increased solubility, and surface reactivity of nanoparticles result in greater and easier nutrient absorption by plants [17]. According to Ahmed et al. [66], foliar spraying of ZnO-NPs had the most impactful outcomes in terms of optimal planting factors, such as plant height, early flowering, fruit yields, and lycopene content. In the present study, it was demonstrated that cherry tomato plants sprayed with ZnO-NPs (3000 ppm) and bioprimed with *T. harzianum* (*T*_h_) had the greatest values of plant length, plant height, and number of leaves per plant with and/or without *F. solani*-infested soil at 75 DAS under greenhouse conditions. In addition, as compared to untreated, infected control plants, it was the most effective interaction treatment for lowering the cherry tomato disease severity index (23.4%), improving the chlorophyll content (SPAD unit) and micronutrient contents (Mn, Zn, and Fe in leaves), and stimulating the phenylalanine ammonia-lyase (PAL) activity of cherry tomato leaves at 75 DAS. The growth-promoting effects of zinc oxide as a nano-fertilizer can be attributed to the activity of Zn, which is an essential micronutrient that is mandatory for optimal plant growth, development, and the biosynthesis of tryptophan and auxins that stimulate cell division, elongation, and the activation of antioxidant enzymes useful in responses to pathogen attacks [15,16]. On the other hand, direct elemental defense by Zn suggests that a high Zn content in plant tissues is more poisonous to the pest/pathogen than to the plant [64,67]. Applications of nanotechnology are considered crucial, less risky, and more useful than those of a variety of other technologies, including genetically engineered technologies [17]. However, it is necessary to conduct a trustworthy risk–benefit analysis and create trustworthy methodologies for assessing the effect of nanomaterials on the environment and human health [17].

Plants respond to microbial pathogen attacks by triggering a variety of defense responses, which are linked to the buildup of defense-related enzymes and inhibitors that aid in pathogen infection prevention [68,69]. PAL is the primary enzyme in the phenylpropanoid pathway, which converts l-phenylalanine to *trans*-cinnamic acid while eliminating ammonia [68,69]. PAL has been shown to be involved in the metabolic activities of many higher plants, and is a crucial enzyme in the synthesis of various defense-related secondary compounds such as phenols and lignins [68,69,70]. The presence of phenolic compounds in plants, as well as their production in response to infection, is linked to disease resistance [68,69,70,71]. According to Vanitha et al. [69], PAL is an inducible enzyme that responds to both biotic and abiotic stresses. It is also regarded as a biochemical marker for plant stress tolerance [69]. Finally, cherry tomato plants can acquire resistance to *Fusarium* wilt caused by *F. solani*, while simultaneously stimulating cherry tomato growth and development in the greenhouse, by biopriming cherry tomato seeds with antagonistic *Trichoderma* isolates and employing foliar spraying of ZnO-NPs, in order to maintain adequate Zn levels in cherry tomato tissues. However, further field studies are needed to investigate the potential of these treatments for controlling *Fusarium* wilt disease caused by *F. solani* before they may be utilized in practice.

## 4. Materials and Methods

### 4.1. Experiment Location and Fungal Strains of the Pathogen and Antagonists

The current research was conducted in-vitro and under greenhouse conditions (GPS: Latitude 31.206134: Longitude 29.919707) located at the Plant Pathology Department, Faculty of Agriculture, Alexandria University, Egypt.

**Figure 9:**
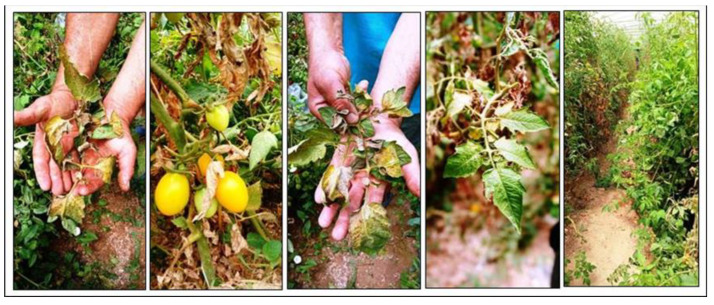
Natural *Fusarium* wilt disease infection of tomato plants in the field at a private farm in Borg El-Arab, Alexandria governorate, Egypt.

We used three fungal species; one fungal pathogen was previously isolated from naturally infected tomato plants, as shown in Figure 9, and identified as *Fusarium solani* (GenBank accession no. OP106576), and two antagonistic *Trichoderma* isolates were previously isolated from soil rhizosphere samples and identified as *Trichoderma harzianum* (*T*_h_) and *Trichoderma viride* (*T*_v_) (GenBank accession numbers: OP106577 and OP106578, respectively), according to Awad-Allah et al. [10].

### 4.2. Evaluation of the Antifungal Activity of ZnO-NPs against F. solani In-Vitro

#### 4.2.1. Culture Medium Amended with ZnO-NPs

Zinc oxide nanoparticles (ZnO-NPs), (30 ± 5 nm particles size, spherical shape, white/light yellow powder, purity 99.9%, formula weight 81.38 g/mol), were purchased from Nano-Gate Company, Cairo, Egypt. In glass tubes, ZnO-NP suspensions were prepared with 10 mL of sterile deionized water, and the corresponding concentrations of nanoparticles were added. The nanoparticles’ suspensions were placed into an ultrasonic liquid processor (BANDELIN electronic, Berlin, Germany, 20 kHz) for 30 min to disrupt the aggregations of the nanoparticles. The culture medium potato-dextrose agar (PDA, HiMedia, MH096-500 G, India) was prepared and used to create a series of media containing ZnO-NPs at concentrations of 0, 250, 500, 1000, 1500, and 3000 mg L^−1^. After that, the autoclaved PDA media with ZnO-NPs at concentrations of 0, 250, 500, 1000, 1500, and 3000 mg L^−1^ were stirred for 15–20 min to achieve homogeneity before each treatment was poured into Petri plates (9 cm in diameter). *F. solani* (Accession no. OP106576) had previously been isolated and molecularly identified [10]. A 5 mm plug of *F. solani* was taken from the edge of a 7-day-old plate, placed in the center of each Petri dish, and incubated in a growth chamber for 12 days at 25 ± 2 °C, according to the modified method described by Sardella et al. [64]. The antifungal activity of ZnO-NP treatments against *F. solani* was evaluated at 2, 4, 6, 8, 10, and 12 days intervals by measuring the diameter of fungal colonies. All experiments were carried out in triplicate, and the diameters were measured in centimeters. The percentage of mycelial growth inhibition was calculated after incubation for 12 days at 25 ± 2 °C using the formula described by Awad-Allah et al. [10]. The percent of inhibition of radial growth (PIRG) = (C − T)/C × 100, where C represents the average value of the radius of the fungus growth (control), and T is the average value of the radius of the inhibited colony (treatment).

Based on the results obtained from this experiment, four treatments (0, 250, 1500, and 3000 ppm) of ZnO-NPs were chosen for evaluation in the greenhouse on cherry tomato plants.

#### 4.2.2. Scanning Electron Microscopy (SEM) Analysis

A scanning electron microscope (SEM) was used to examine the morphological changes of *F. solani* hyphae before and after ZnO-NP treatments, according to the modified method described by Sardella et al. [64]. Pieces of mycelial material were cut from 7-day-old cultures and inoculated onto PDA containing 3000 mg L^−1^ ZnO-NPs in comparison with a control containing no ZnO-NPs, followed by a 12-day incubation period at 25 ± 2 °C. After that, pieces of mycelia were cut from the edge of the fungal cultures and directly analyzed at different magnifications using the JSM-IT200 SEM series (JEOL Ltd., Tokyo, Japan) at the Electron Microscope Unit, Faculty of Science, Alexandria University, Alexandria, Egypt.

### 4.3. Identification of Secondary Metabolites of Trichoderma Isolates by Gas Chromatography–Mass Spectrometry (GC–MS) Analysis:

#### 4.3.1. Extraction of Bioactive Secondary Metabolites (SMs)

The abilities of two antagonistic *Trichoderma* isolates (*T*_h_ and *T*_v_) to produce antifungal secondary active metabolites were tested and identified using GC–MS analysis. For the extraction of secondary metabolites (SMs), two 7 mm diameter discs of each *Trichoderma* isolate (*T*_h_ and *T*_v_) were taken from actively growing margins of PDA cultures and separately inoculated into 2 L conical flasks containing 250 mL of pre-sterilized potato dextrose broth (PDB, HiMedia, India). The suspension cultures were incubated for 10 days at 28 °C, after which the fungal mycelium was filtered out of the broth according to the modified method described by Vinale et al. [72]. The filtered cultures broth of *Trichoderma* isolates were extracted twice with equal volumes of ethyl acetate (EtOAc). The ethyl acetate layer containing SMs was separated, and the volatile compounds of secondary metabolites were identified using a GC–MS technique.

#### 4.3.2. GC–MS Analysis of Secondary Metabolites

The chemical compositions of the crude ethyl acetate extracts of *Trichoderma* isolates were analyzed using a Trace GC-TSQ mass spectrometer (Thermo Scientific, Austin, TX, USA) with a direct capillary column TG–5MS (30 m × 0.25 mm × 0.25 µm film thickness). The column oven temperature was initially kept at 50 °C before increasing by 5 °C per minute to 250 °C for 2 min. Then, the temperature was raised by 30 °C per minute to the final temperature of 300 °C before being held for 2 min. Helium was used as a carrier gas at a constant flow rate of 1 mL/min, with the injector and MS transfer line maintained at constant temperatures of 270 and 260 °C, respectively. Using an Autosampler AS1300 connected to a GC operating in split mode, diluted samples containing 1 µL were automatically injected with a solvent delay of 3 min. EI mass spectra were collected at 70 eV ionization voltages over the range of *m*/*z* 50–650 in full scan mode. The temperature of the ion source was set at 200 °C. The components were identified by comparing their mass spectra with those of WILEY (Wiley Registry of Mass Spectral Data, 9th Edition Version 1.02) and NIST 14 (NIST/EPA/NIH mass spectral library) mass spectral databases, as described by Abd El-Kareem et al. [73].

### 4.4. Effects of Seed-Biopriming with Trichoderma and ZnO-NPs against Tomato Fusarium Wilt Disease under Greenhouse Conditions

#### 4.4.1. Fungal Inoculum Preparation of the Pathogen

The pathogenic cultures of *F. solani* were brought from the Department of Plant Pathology, Faculty of Agriculture, Alexandria University. In brief, the fungal inoculum was cultured onto PDA plates and incubated for 10 days at 25 ± 1 °C in 90 mm Petri plates. According to Awad-Allah et al. [10], after incubation, the plates were filled with sterile water and the conidia were scraped using a sterile glass rod. After filtering the spore solutions to eliminate fungal hyphae and conidial residue, the conidial suspension concentration was adjusted to 1 × 10^7^ conidia mL^−1^ using a hemocytometer. For the greenhouse experiment, the inoculum concentration of *F. solani* was adjusted to 10^3^ conidia g^−1^ soil.

#### 4.4.2. Preparation of Trichoderma Spore Suspensions for Seed-Biopriming

The cultures of *T. harzianum* (*T*_h_) and *T. viride* (*T*_v_) were individually grown onto PDA plates and incubated for 10 days at 25 ± 1 °C. After incubation, the fungal spores were harvested in sterile distilled water and filtered with sterile muslin cloth, according to Awad-Allah et al. [10]. The spore concentration was adjusted to 1 × 10^7^ conidia mL^−1^ using a hemocytometer, followed by centrifugation at 10,000 rpm for 10 min. The pellet was re-suspended in the same volume of autoclaved 1.5% Carboxy methyl cellulose (CMC) solution, which acts as an adherent during priming according to the adapted method described by Singh et al. [9].

For seed-biopriming with a spore suspension of *T. viride* (*T*_v_) and *T. harzianum* (*T*_h_), healthy seeds of cherry tomato (*Solanum lycopersicum* L.; Huang Sheng “Golden Cherry” HYBRID F1, China) were surface sterilized with 1% sodium hypochlorite (NaOCl) for 1 min, rinsed three times for 5 min with autoclaved distilled water, and surface-dried under laminar air flow for about 2 h, according to Jain et al. [74]. The surface-sterilized and dried seeds were treated by soaking for 30 min in the aforementioned spore suspensions of *T. viride* (*T*_v_) and *T. harzianum* (*T*_h_), and the control seeds (*T*_0_) were treated with only an autoclaved 1.5% CMC solution without spore suspension. After priming, the excess suspension was drained out, and all treated seeds were put in an incubator at 28 ± 2 °C with 98% relative humidity for 24 h before being surface-dried under sterile air in laminar air flow for 2 h, according to Jensen et al. [75].

#### 4.4.3. Pot Trials, Treatments, and Experimental Design

Pot trials were conducted in a controlled greenhouse at an average temperature of 25 ± 2 °C, a humidity of 77 ± 5%; and a photoperiod of 14/10 h of light/dark. Plastic pots (20 cm inner diameter) were filled with a sterilized mixture (2:1 *v*/*v*) sandy loam–peat moss (NORD AGRI SIA, Riga, Latvia). The soil was artificially infected with *F. solani* by adding (10^3^ conidia g^−1^ soil) conidial suspension and kept for 7 days with regular irrigation for pathogen establishment.

The trial treatments were arranged using a Randomized Complete Block Design (RCBD), with five replicates for each treatment. This study included twenty-four treatments, including two pathogenic fungal treatments (with and/or without *F. solani*-infested soil) and all combinations of three seed-priming treatments of antagonistic *Trichoderma* isolates (*T*_0_, *T*_v_, and *T*_h_) with four foliar spraying of ZnO-NP aqueous solutions (0, 250, 1500, and 3000 ppm).

In a controlled environment, bioprimed cherry tomato seeds with different *Trichoderma* spore suspensions (*T*_0_, *T*_v_, and *T*_h_) were sown into soil pots (i.e., either with and/or without *F. solani* infested soil pots) on 15 September 2022. These seeds were watered daily with autoclaved distilled water, and two weeks after sowing, the seedlings were thinned to three plants per pot. The 15-day-old cherry tomato seedlings were irrigated with a half-strength, Zn-free nutrient solution of Hoagland and Arnon as a base solution at a pH of 5.5–6.0, according to Hewitt [76], using a surface drip irrigation system in the greenhouse. Foliar treatments of ZnO-NPs (0, 250, 1500, and 3000 ppm) were applied to cherry tomato leaves by spraying 200 mL per plant of each concentration using a hand-held sprayer bottle at 30, 45, and 60 days after sowing (DAS), whereas control plants were sprayed with autoclaved distilled water. Five drops of 80% Tween^®^ 20 were added to each prepared solution to maximize dissemination on cherry tomato leaves. Cherry tomato plants per treatment were grown, and the experiment was terminated at 75 DAS.

### 4.5. Measurements

#### 4.5.1. Disease Severity Index (DSI)

The disease was assessed using the severity scale of 0 to 4 based on the presence or absence of symptoms, with zero denoting no chlorosis or wilt symptoms for a healthy plant, one denoting the first symptoms of chlorosis of the leaves, two denoting severe chlorosis of the leaves and initial symptoms of wilting, three indicating serious symptoms of wilting and chlorosis of the leaves, and four denoting a plant that is totally withered and completely necrotic, according to Vega-Gutiérrez et al. [77]. The Disease Severity Index (DSI) was calculated with the formula proposed by Vega-Gutiérrez et al. [77].

$$DSI\left(\%\right)=\sum [P\times Q]/[M\times N]\times 100$$

where *P* is the severity point, *Q* is the number of plants infected with some scale, *M* is the total number of plants observed, and *N* is the maximum classification in the number of the scale.

#### 4.5.2. Vegetative Growth Parameters

Five cherry tomato plants were randomly selected from each treatment at 75 DAS to assess vegetative growth parameters, i.e., plant length (cm), plant height (cm), and number of leaves per plant.

#### 4.5.3. Mineral Content in Cherry Tomato Leaves

The newest fully-expanded cherry tomato leaves were sampled and oven dried at 70 °C for 48 h, and the levels of three essential micronutrients such as manganese, zinc, and iron contents were measured, according to Jones Jr. [78].

#### 4.5.4. Leaf Chlorophyll Content (SPAD Value)

Total chlorophyll content was measured as a SPAD value, and the portable chlorophyll meter SPAD-502 Plus (Konica-Minolta, Inc., Tokyo, Japan) was used.

#### 4.5.5. Phenylalanine Ammonia-Lyase (PAL) Activity Measurements

PAL activity was determined at 30 °C by direct spectrophotometric measurement as the rate of conversion of L-phenylalanine to *trans*-cinnamic acid at 290 nm, as reported by Dickerson et al. [79]. One gram of fresh leaves was homogenized in 5 mL of 0.1 M sodium borate buffer (pH 7.0) containing 0.1 g of insoluble polyvinylpyrrolidone (PVP). The extract was filtered through cheesecloth, and the filtrate was centrifuged at 20,000 g for 35 min at 4 °C. The supernatant was used as an enzyme source. Samples containing 0.4 mL of enzyme extract were incubated with 0.5 mL of 0.1 M borate buffer, pH 8.8, and 0.5 mL of 12 mM L-phenylalanine in the same buffer for 30 min at 30 °C. In the reference cell, 0.4 mL of enzyme extract was taken along with 1.0 mL of borate buffer. The amount of *trans*-cinnamic acid synthesized was calculated using its extinction coefficient of 9630 M^−1^ cm^−1^. Enzyme activity was expressed as the synthesis of *trans*-cinnamic acid (nmol. min^−1^·g^−1^ of fresh weight).

### 4.6. Statistical Analysis

The in-vitro and greenhouse experimental studies were repeated twice, an analyzed data of variance (ANOVA) was carried out for each experimental study separately, and a combined analysis was conducted throughout each repeated experimental study due to the homogeneity of the error variance. All statistical tests were performed using the CoStat statistical analysis program [80] (CoHort Software version 6.303, Monterey, CA, USA), the analyzed data of variance (ANOVA) according to Gomez and Gomez [81], and least significant difference (LSD) at significance levels of *(p* ≤ 0.05) or (*p* ≤ 0.01) were employed for comparison of means. The in-vitro investigation used three replications, and two-way analyses of variance (ANOVA) were used to examine the effects of time and culture medium amended with different concentrations of ZnO-NPs on *F. solani* colony diameter (cm). Mycelial growth inhibition (%) was presented as mean ± standard deviation (mean ± SD). In the greenhouse trials, five replications were used. Two-way ANOVA tests were used on the data of disease severity index (%) of infected cherry tomato plants to examine the individual and combined effects of seed-biopriming with *Trichoderma* isolates and foliar spray of ZnO-NPs. The data on vegetative growth parameters, mineral content in cherry tomato leaves, leaf chlorophyll content (SPAD value), and PAL activity were subjected to three-way analyses of variance (ANOVA) to examine the effects of seed-biopriming with *Trichoderma* isolates and foliar spray of ZnO-NPs with and/or without *F. solani*-infested soil and their interactions on various variables.

## 5. Conclusions

The in-vitro antifungal activity of ZnO-NPs was shown to be concentration-dependent. As a result, the highest treatment (3000 ppm) of ZnO-NPs had the greatest reduction of *F. solani* mycelial growth. SEM investigation revealed apparent deformation in mycelia treated with ZnO-NPs, which might be the source of mycelial growth suppression. In addition, we isolated and identified twenty-eight bioactive compounds from two *Trichoderma* spp. ethyl acetate crude extracts, all of which have strong antimicrobial and plant growth-promoting activities. In a greenhouse, seed-biopriming with *Trichoderma* isolates promotes their proliferation on the seed surface, which may help in the secretion of different arrays of bioactive compounds surrounding seeds, and thus can protect seeds from rhizosphere pathogens. Additionally, ZnO-NPs have shown increased antifungal efficacy when combined with seed-biopriming treatments. Foliar application of ZnO-NPs reduced the severity of *Fusarium* wilt, allowing the growth of bioprimed cherry tomatoes. Also, ZnO-NPs have the ability to act as a biostimulant to improve vegetative growth parameters, micronutrient levels (Mn, Zn, and Fe in leaves), chlorophyll content, and PAL activity in bioprimed cherry tomato leaves.

## Figures and Tables

**Table 1:** Effect of ZnO-NPs on the colony diameter of *F. solani* incubated for 12 days at 25 ± 2 °C in-vitro test.

ZnO-NPs (ppm)	Colony Diameter (cm)							Mean
	Time (Day)							
	0	2	4	6	8	10	12	
0	^¶^0.5 ± 0.01 ^v^	2.8 ± 0.10 ^mn^	5.3 ± 0.17 ^hi^	7.7 ± 0.20 ^c^	8.1 ± 0.14 ^b^	8.6 ± 0.20 ^a^	8.8 ± 0.13 ^a^	6.0 ± 3.08 ^a^
250	0.5 ± 0.02 ^uv^	1.4 ± 0.01 ^r^	3.0 ± 0.05 ^lm^	5.3 ± 0.05 ^h^	6.0 ± 0.50 ^f^	6.7 ± 0.50 ^e^	7.3 ± 0.40 ^d^	4.3 ± 2.56 ^b^
500	0.5 ± 0.01 ^v^	1.0 ± 0.02 ^s^	2.4 ± 0.05 ^o^	3.6 ± 0.20 ^k^	4.4 ± 0.40 ^j^	5.0 ± 0.40 ^i^	5.7 ± 0.40 ^g^	3.2 ± 1.91 ^c^
1000	0.5 ± 0.02 ^uv^	0.9 ± 0.01 ^st^	2.1 ± 0.01 ^p^	2.6 ± 0.01 ^no^	3.1 ± 0.05 ^l^	3.6 ± 0.10 ^k^	4.2 ± 0.10 ^j^	2.4 ± 1.29 ^d^
1500	0.5 ± 0.01 ^v^	0.8 ± 0.05 ^stuv^	1.3 ± 0.01 ^r^	1.4 ± 0.02 ^r^	1.8 ± 0.01 ^q^	2.1 ± 0.01 ^p^	2.4 ± 0.10 ^o^	1.4 ± 0.65 ^e^
3000	0.5 ± 0.01 ^v^	0.5 ± 0.02 ^uv^	0.5 ± 0.01 ^v^	0.6 ± 0.02 ^tuv^	0.6 ± 0.03 ^uv^	0.7 ± 0.03 ^tuv^	0.8 ± 0.02 ^stu^	0.6 ± 0.11 ^f^
Mean	0.5 ± 0.01 ^g^	1.2 ± 0.77 ^f^	2.4 ± 1.55 ^e^	3.5 ± 2.48 ^d^	4.0 ± 2.60 ^c^	4.4 ± 2.76 ^b^	4.9 ± 2.84 ^a^	
L.S.D. _0.05_	ZnO-NPs = 0.1126			Time = 0.1216		ZnO-NPs × Time = 0.2979		
*F*-value	ZnO-NPs = 2380.97 *			Time = 1475.28 *		ZnO-NPs × Time = 132.91 *		
*C.V.*	5.01%							

Means followed by the same alphabetical letter(s) are not significantly different at *p* ≤ 0.05 (*). ^¶^ Values are expressed as mean ± SD (n = 3). *C.V.*, coefficient of variation (%).

**Table 2:** Identification list of bioactive secondary metabolites of *T. viride* and *T. harzianum* detected by GC–MS; a ≥ 65% match quality in the Wiley Registry^®^ of Mass Spectral Data Library.

Compound Name	*T. viride*		*T. harzianum*		Molecular Formula	Cas #	Biological Properties	Literature(s)
	RT (min)	Area (%)	RT (min)	Area (%)				
Strychane, 1-acetyl-20á-hydroxy-16-methylene	4.63	1.26	ND		C_21_H_26_N_2_O_2_	2111-98-0	Antimicrobial activities	[18]
2-Aminoethanethiol hydrogen sulfate (ESTER)	4.63	0.63	ND		C_2_H_7_NO_3_S_2_	2937-53-3	Stimulatory/nutritive effect; antioxidant; antibacterial; antiviral activities	[19,20]
2,4-Di-tert-butylphenol	8.19	5.80	8.18	7.09	C_14_H_22_O	96-76-4	Plant growth promoter; antioxidant; antibacterial; protective; curative activities	[21,22,23]
Phenol, 3,5-bis(1,1-dimethylethyl)-	9.16	4.73	9.16	5.85	C_14_H_22_O	1138-52-9	Antioxidant; anti-proliferative; antimicrobial; cytotoxic activities	[24,25]
1-Hexadecanol, 2-methyl-	11.12	4.26	11.11	5.18	C_17_H_36_O	2490-48-4	An elicitor for systemic acquired resistance; antimicrobial; antioxidant	[26,27,28,29]
Nonadecane	ND		11.95	2.18	C_19_H_40_	629-92-5	Antifungal activity	[30,31]
Methoxyacetic acid, 2-tetradecyl ester	12.03	4.74	12.03	6.45	C_17_H_34_O_3_	N/A	Cytotoxic; antimicrobial; antifungal activities	[32,33]
1,3,5-Triazine-2,4-diamine, 6-chloro-N-ethyl-	12.58	16.02	12.58	18.87	C_5_H_8_ClN_5_	1007-28-9	An elicitor for systemic acquired resistance; growth stimulator; antifungal activity	[28,34,35]
tert-Hexadecanethiol	13.14	1.31	ND		C_16_H_34_S	25360-09-2	Antibacterial; antifungal; antibiotic activities	[36,37]
Heptadecane, 2,6,10,15-tetramethyl-	13.47	14.11	13.47	20.99	C_21_H_44_	54833-48-6	Growth stimulator; antioxidant; antimicrobial	[38,39,40]
1,2-15,16-Diepoxyhexadecane	ND		14.09	4.26	C_16_H_30_O_2_	N/A	Antioxidant; antimicrobial; antifungal actions	[41,42,43]
17-Octadecynoic acid	ND		14.09	0.58	C_18_H_32_O_2_	34450-18-5	Antimicrobial; antifungal; antibiotic activities	[44,45]
Cholestan-3-ol, 2-methylene-, (3á,5à)-	ND		14.09	0.58	C_28_H_48_O	22599-96-8	Antibacterial; antifungal; antiviral; anti-oxidative activities	[46,47]
Z-10-Methyl-11-tetradecen-1-ol propionate	ND		14.85	3.58	C_18_H_34_O_2_	N/A	Antioxidant; antiviral; antimicrobial activities	[48]
2,2,3,3,4,4 hexadeutero octadecanal	15.25	5.50	15.24	7.03	C_18_H_30_D_6_O	56554-51-9	An elicitor for systemic acquired resistance; antimicrobial; antioxidant; cytotoxic activities	[28,49]
7-Methyl-Z-tetradecen-1- ol acetate	ND		15.57	3.51	C_17_H_32_O_2_	N/A	Antimicrobial; antifungal activity	[29,50]
11,14-Eicosadienoic acid, methyl ester	ND		15.81	4.54	C_21_H_38_O_2_	2463-02-7	Antifungal; antioxidant activities	[51,52]
2,2-Dideutero Octadecanal	ND		16.08	3.92	C_18_H_34_D_2_O	56555-07-8	Antioxidant; antibacterial; cytotoxic activities	[53,54]
Ethanol, 2-(Octadecyloxy)	ND		16.47	2.65	C_20_H_42_O_2_	2136-72-3	Antifungal; antimicrobial activities	[42,55]
Tetradecane, 2,6,10-trimethyl-	16.61	21.70	16.61	22.88	C_17_H_36_	14905-56-7	Antioxidant; antimicrobial activities	[43,54]
Octadecane, 3-ethyl-5-(2-ethylbutyl)-	17.39	5.91	17.38	8.02	C_26_H_54_	55282-12-7	Nematicidal property; antimicrobial; antiviral activities	[56,57,58]
Aspidospermidin-17-ol, 1-acetyl-19,21-epoxy-15,16-dimethoxy-	ND		18.39	1.08	C_23_H_30_N_2_O_5_	2122-26-1	Antioxidant; antibacterial; anti-proliferative activities	[59,60]
Octadecane, 1-chloro-	20.24	11.57	20.24	16.28	C_18_H_37_Cl	3386-33-2	Antimicrobial; antibiotic activities	[61]
Limonen-6-ol, pivalate	20.64	0.48	ND		C_15_H_24_O_2_	N/A	Antibacterial; antimicrobial activities	[62]
Decane, 2,3,5,8-tetramethyl-	23.53	9.48	23.53	13.07	C_14_H_30_	192823-15-7	Antimicrobial; antibiotic activities	[61]
2-(3,4-dimethoxyphenyl)-3,5-dihydroxy-7-methoxy-4H-1-Benzopyran-4-one	26.53	3.99	26.53	5.01	C_18_H_16_O_7_	6068-80-0	Antioxidant; antimicrobial properties	[63]
Hexadecanoic acid, ethyl ester	ND		29.24	0.50	C_18_H_36_O_2_	N/A	Antiviral; antimicrobial activities	[57]
9-Octadecenoic acid (Z)-, methyl ester	ND		31.77	0.53	C_19_H_36_O_2_	112-62-9	Antioxidant; antibacterial; anti-proliferative; antimicrobial activity	[42,60]

RT: retention time; ND: not detected: N/A: not available; Cas: chemical abstracts service; Cas #: Cas registry number.

**Table 3:** Effects of different treatments of foliar spray of ZnO-NPs and seed-biopriming with *Trichoderma* isolates on the disease severity index (%) of infected cherry tomato plants grown for 75 DAS under greenhouse conditions.

ZnO-NPs (ppm)	Disease Severity Index (%)			Mean
	Seed-Biopriming with *Trichoderma* Isolates			
	*T*_0_	*T*_v_	*T*_h_	
0	^¶^92.6 ± 3.75 ^a^	81.0 ± 0.71 ^b^	75.4 ± 1.67 ^c^	83.0 ± 7.75 ^a^
250	80.2 ± 2.68 ^b^	70.4 ± 1.67 ^d^	60.0 ± 0.71 ^f^	70.2 ± 8.71 ^b^
1500	63.4 ± 2.88 ^e^	55.0 ± 0.71 ^g^	40.0 ± 0.71 ^i^	52.8 ± 10.15 ^c^
3000	45.2 ± 2.68 ^h^	33.0 ± 0.71 ^j^	23.4 ± 1.47 ^k^	33.9 ± 9.38 ^d^
Mean	70.4 ± 18.52 ^a^	59.9 ± 18.54 ^b^	49.7 ± 20.24 ^c^	
L.S.D. _0.01_	ZnO-NPs = 1.9349	Seed-biopriming = 1.6757	ZnO-NPs × Seed-biopriming = 3.3514	
*F*-value	ZnO-NPs = 1765.37 **	Seed-biopriming = 550.43 **	ZnO-NPs × Seed-biopriming = 5.34 **	
*C.V.*	3.28%			

Means followed by the same alphabetical letter(s) in common are not significantly different at *p* ≤ 0.01 (**). ^¶^ Values are expressed as mean ± SD (n = 5). *C.V.*, coefficient of variation (%).

## Data Availability

The data presented in this study are available on request from the corresponding author.
